# Response to “Reflection on the Integration of Artificial Intelligence in Anaesthesiology: Beyond Algorithmic Performance”

**DOI:** 10.4274/TJAR.2026.262502

**Published:** 2026-04-15

**Authors:** Burhan Dost, Engin İhsan Turan, Muhammed Enes Aydın, Ali Ahıskalıoğlu, Madan Narayanan, Resul Yılmaz, Alessandro De Cassai

**Affiliations:** 1 Ondokuz Mayıs University Faculty of Medicine, Department of Anaesthesiology and Reanimation, Samsun, Türkiye; 2 University of Health Sciences Türkiye, İstanbul Kanuni Sultan Süleyman Training and Education Hospital, Clinic of Anaesthesiology and Reanimation, İstanbul, Türkiye; 3 Atatürk University Faculty of Medicine, Department of Anaesthesiology and Reanimation, Erzurum, Türkiye; 4 Frimley Park Hospital, NHS Frimley Health Foundation Trust, Camberley, UK; 5 Necmettin Erbakan University Faculty of Medicine Hospital, Department of Anaesthesiology and Reanimation, Konya, Türkiye; 6 University of Padua (DIMED), Department of Medicine, Padua, Italy; 7 University Hospital of Padua, Institute of Anaesthesia and Intensive Care Unit, Padua, Italy

**Keywords:** Artificial intelligence, hypotension prediction index, perioperative care

## Dear Editor,

We sincerely thank the authors for their thoughtful and insightful letter^1^ regarding our review.^2^ We appreciate their careful reading of our work and constructive reflections on the evolving role of artificial intelligence (AI) in anaesthesiology.

We fully agree that a critical challenge lies in bridging the gap between the algorithmic performance and real-world clinical benefits. As highlighted in both our review and the authors’ letter, high discriminative accuracy does not necessarily translate to improved patient-centred outcomes. The example of the hypotension prediction index (HPI) is particularly illustrative: despite promising predictive metrics, the relatively low positive predictive value in clinical settings raises valid concerns regarding alarm fatigue, cognitive overload, and the potential for inappropriate therapeutic interventions.^3^ We concur that future investigations must increasingly prioritise outcome-driven, pragmatic clinical trials rather than surrogate technical endpoints alone. Despite these concerns, the evidentiary landscape surrounding HPI continues to evolve and extend beyond early validation cohorts. The recently published randomised controlled trial protocol by Mulder et al.^4^, which compared a conventional mean arterial pressure alarm strategy with HPI-guided management in moderate- to high-risk non-cardiac surgical patients within a non-inferiority framework, directly examined whether predictive waveform analytics provide incremental clinical value beyond traditional threshold-based monitoring. In parallel, emerging data from critically ill populations, such as the prospective study by Khwannimit et al.^5^ evaluating HPI performance in patients with septic shock in the intensive care unit, demonstrate both the potential and present limitations of the algorithm in high-risk settings. Taken together, these investigations highlight a broader physiological reality: haemodynamic instability is complex and dynamic and is shaped by multiple interacting variables. No single measurement can fully represent this complexity of the disease. Therefore, HPI should not be interpreted as a replacement for conventional monitoring but rather as a contributory element within an evolving multimodal decision-support framework that integrates pressure trends, waveform characteristics, clinical context, and physician judgment.

We also strongly support the authors’ emphasis on the educational implications of their findings. The integration of AI into regional anaesthesia, airway management, and haemodynamic monitoring should not replace fundamental clinical reasoning or anatomical expertise. In contrast, we believe that these systems should be designed to function as supervised educational tools that reinforce decision-making, pattern recognition, and situational awareness. Safeguarding against clinical deskilling must remain a core objective as AI becomes increasingly embedded in anaesthesia training programs.

Furthermore, we appreciate the important points raised regarding equity, generalisability, and dataset bias. The predominance of training data from high-income healthcare systems may limit the external validity of many AI models. Broader, multicentre, and internationally diverse validation studies are essential to prevent the emergence of unequal standards of care and ensure that AI-based tools remain reliable across heterogeneous patient populations and clinical environments.

In addition, the integration of large language models into clinical practice requires evaluation beyond technical performance, incorporating considerations of safety, clinical relevance, and responsible implementation.^6^ The authors highlight that high accuracy alone does not guarantee clinical benefit and that AI-generated outputs must be interpreted within the clinical context, under human supervision, and through structured evaluation processes. They further stressed that these systems may play an important role as supportive tools in medical education but should not replace fundamental clinical reasoning or expertise. This perspective closely aligns with the principles highlighted in our review, namely that AI in anaesthesiology should be positioned as an adjunct that strengthens patient safety, educational quality, and clinical decision-making, rather than as a substitute for clinical judgment.

In conclusion, we are grateful to the authors for expanding the discussion beyond technical capability to clinical responsibility. We fully agree that the next phase of AI research in anaesthesiology must focus on patient-centred outcomes, external validation, educational safeguards, and transparent accountability frameworks.
